# State of the Patients Admitted at the Bristol Dispensary, from the 1st of December, 1801, to the 1st of January, 1802

**Published:** 1802-04

**Authors:** W. D. Rolfe

**Affiliations:** Dispensary House


*State of the Patients admitted at the Bristol Dispensary, from the 1st of December,* 1801, *to the 1st of January,* 1802.

| | | | |
|---|---|---|---|
| Asthma | 3 | Menorrhagia | 1 |
| Anasarca | 1 | Nervous | 1 |
| Ascites | 1 | Pleuritis | 5 |
| Cynanche Tonsillaris | 2 | Phthisis Pulmonalis | 3 |
| ——— Maligna | 1 | Peripneumonia | 6 |
| Consumption | 4 | Pertussis | 3 |
| Dysenteria | 5 | Rheumatismus Acutus | 1 |
| Diarrhœa | 1 | Scrophula | 3 |
| Dyspepsia | 3 | Scorbutic | 1 |
| Erysipelas | 2 | Synocha | 14 |
| Febris Puerperalis | 1 | Typhus | 16 |
| Gastrodynia | 2 | ——— Putrida | 1 |
| Gravel | 1 | Tussis | 2 |
| Hydrothorax | 1 | Tinea | 1 |
| Ischuria | 2 | Variola Putrida | 1 |
| Lumbago | 1 | | |

| | | |
|---|---|---|
| Patients admitted in the course of last year | | 1353 |
| Cured | 1108 | |
| Relieved | 45 | |
| Died (many of whom where in the last stage of consumption) | 103 | |
| Remain sick | 97 | |
| Total of sick patients in twenty-six years | | 15135 |
| Midwifery patients delivered at their own houses this year | | 309 |
| Children born { Boys 166, Girls 148 } | 314 | |
| Remain undelivered | | 67 |
| Total of midwifery patients in twenty-four years | | 4884 |
| Children born { Boys 2545, Girls 2379 } | | |
| Total, in 24 years, | 4924 | |

*Dispensary House, Jan.* 9, 1802.

W. D. ROLFE.

